# Production Performance, Nutrient Digestibility, and Milk Composition of Dairy Ewes Supplemented with Crushed Sunflower Seeds and Sunflower Seed Silage in Corn Silage-Based Diets

**DOI:** 10.3390/ani10122354

**Published:** 2020-12-09

**Authors:** Eduardo Cardoso-Gutiérrez, Alondra Cristel Narváez-López, Lizbeth E. Robles-Jiménez, Andrés Morales Osorio, María de Guadalupe Gutierrez-Martinez, Heidi Leskinen, Marcello Mele, Einar Vargas-Bello-Pérez, Manuel González-Ronquillo

**Affiliations:** 1 Facultad de Medicina Veterinaria y Zootecnia, Instituto Literario 100, Universidad Autónoma del Estado de México, Toluca CP 50000, Mexico; cardosoge89@gmail.com (E.C.-G.); lizroblez@hotmail.com (L.E.R.-J.); 2 Division Académica de Ciencias Agropecuarias, Universidad Juárez Autónoma de Tabasco, Carr. Villahermosa-Teapa, km 25, Villahermosa, Tabasco CP 86280, Mexico; londycristel1996@gmail.com; 3 Facultad de Ciencias Agricolas, Instituto Literario 100, Universidad Autónoma del Estado de México, Toluca CP 50000, Mexico; amosorio47@yahoo.com.mx (A.M.O.); mggutierrezm@uaemex.mx (M.d.G.G.-M.); 4 Milk Production, Production Systems, Natural Resources Institute Finland (Luke), FI 31600 Jokioinen, Finland; heidi.leskinen@luke.fi; 5 Dipartimento di Scienze Agrarie, Alimentari e Agro-Ambientali, Università di Pisa, Via del Borghetto 80, 56124 Pisa, Italy; marcello.mele@unipi.it; 6 Department of Veterinary and Animal Sciences, Faculty of Health and Medical Sciences, University of Copenhagen, Grønnegårdsvej 3, 1870 Frederiksberg C, Denmark

**Keywords:** sheep, oilseeds, rumen, digestibility, sunflower

## Abstract

**Simple Summary:**

In countries such as Mexico and the Unites States, sunflowers can be planted earlier than corn, and can tolerate moderate frosts. Concerning sunflower silage, its feeding value could be up to 80% similar to that of corn silage. The use of sunflower silage for ruminant feeding represents an alternative to the use of corn silage, especially when environmental conditions are adverse for corn crops. Sunflower seeds can also be a protein alternative to soybean by-products. This study determined production performance, nutrient digestibility, and milk composition of dairy ewes fed with crushed sunflower seeds (SF) and sunflower seed silage (SFS) in corn silage-based diets. Compared to control, SF and SFS increased intake and digestibility of fiber components, such as neutral detergent fiber (NDF) and acid detergent fiber (ADF). Nitrogen balance, milk yield, milk fat yield, and milk protein yield were similar between treatments. Results demonstrated that crushed sunflower seeds and ensiled seeds do not change significantly productive parameters of dairy sheep. In corn-silage based diets, both crushed and ensiled sunflower seeds can be used in dairy sheep diets as alternatives to typical protein feedstuffs, such as soybean meal.

**Abstract:**

This study determined production performance, nutrient digestibility, and milk composition of dairy ewes supplemented with crushed sunflower seeds (*Helianthus annuus*) and sunflower seed silage in corn silage-based diets. Six ewes were grouped in a double 3 × 3 Latin square design with three periods of 21 days. All treatments were based on *ad libitum* corn silage. Control diet was based on alfalfa hay (333 g/kg DM), sorghum grain (253 g/kg DM), triticale grain (200 g/kg DM), soybean meal (167 g /kg DM), and vitamin and mineral premix (47 g/kg DM). Sunflower seeds (SF) and sunflower seed silage (SFS) treatments consisted of alfalfa hay (333 g/kg DM), sorghum grain (267 g/kg DM), triticale grain (100 g/kg DM), soybean meal (167 g /kg DM), SF or SFS (87 g/kg DM) and vitamin and mineral premix (47 g/kg DM). Compared to control, SF and SFS increased intake and digestibility of fiber components, such as neutral detergent fiber (NDF) and acid detergent fiber (ADF). Body weight, nitrogen balance, milk yield, milk fat yield, milk protein yield, lactose yield and milk urea N were similar between treatments. Overall, results demonstrated that crushed sunflower seeds and ensiled seeds do not change significantly productive parameters of dairy sheep.

## 1. Introduction

After soybean, sunflower is one of the world’s most important oilseed crops used for oil production, and it is high in oleic acid [1]. Sunflower seeds are mostly produced for oil extraction, but can also be used as a protein source for monogastric and ruminant animals, as they provide high contents of fat (380–540 g/kg) and protein (140–200 g/kg) [2].

In countries such as Mexico and the Unites States, sunflowers can be sowed earlier than corn, and can tolerate moderate frosts. Concerning sunflower silage, its feeding value could be up to 80% similar to that of corn silage. The main difference is that whole-plant sunflower silage has more crude protein and fat than corn silage [3]. Moreover, sunflower is usually cultivated under rainfed conditions, whereas corn cultivation is usually associated with high water consumption for irrigation. Therefore, the use of sunflower silage for ruminant feeding represent an alternative to the use of corn silage, especially when environmental conditions are adverse for corn crops. Moreover, oilseed oil by-products, such as sunflower meal and rapeseed meal, could be alternative protein feedstuffs instead of using soybean meal in sheep diets [4,5].

Adding oilseeds to lactating ewe diets can improve milk fatty acid profiles. Zhang et al. [6] described how dietary supplementation with seeds of canola, sunflower, or flaxseed can be used to modify milk fatty acid structure towards a healthier profile for human consumption, without effects on dry matter intake or nutrient utilization. Oilseed by-products have been used in ewe’s diets. For example, supplementation of sunflower oilcake at indoor or outdoor feeding systems has been shown to improve the content of total unsaturated milk fatty acids without deleterious outcomes on milk production and milk composition [7]. In grazing ewes, sunflower seeds have shown to improve the content of polyunsaturated fatty acids without affecting milk yield [8].

Today, no studies are available in the literature that concurrently relate the effects of feeding different sunflower products to dairy ewes. Therefore, the aim of this study was to quantify milk production, nutrient digestibility, and milk composition of dairy ewes supplemented with crushed sunflower seeds and sunflower seed silage in corn silage-based diets. The hypothesis of this study was that the type of sunflower seed supplementation (as a crushed seed or as a seed silage) affects production performance, nutrient digestibility, and milk composition. Our data will be important for sheep farmers looking for alternative energy and protein feedstuffs.

## 2. Materials and Methods

### 2.1. Animal Conditions and Experimental Diets

Animal procedures were approved by the Animal Experimental Guidelines of the Universidad Autonoma del Estado de México (project code UAEMex 4974/2020).

The study was performed at the Universidad Autónoma del Estado de México. Six primiparous East-Friesian ewes with 70 ± 5 days in milk were fed with three diets according to double 3 × 3 Latin square design, with 21 d experimental periods. Each experimental period consisted of 15 d for diet adaptation and 6 d for sample collection. During the study, animals were assigned into individual metabolic cages (1.2 × 0.8 m), fed twice daily (08:00 and 15:00 hours) with continuous water supply, and milked manually, daily, at 16:00 hours.

All treatments contained corn silage ad libitum. Control diet contained alfalfa hay (333 g/kg DM), sorghum grain (253 g/kg DM), triticale grain (200 g/kg DM), soybean meal (167 g /kg DM), and vitamin and mineral premix (47 g/kg DM; Multitec of Malta^®^, Guanajuato, Mexico).

Crushed sunflower seed (SF) and sunflower seed silage (SFS) diets were composed by alfalfa hay (333 g/kg DM), sorghum grain (267 g/kg DM), triticale grain (100 g/kg DM), soybean meal (167 g /kg DM), SF or SFS (87 g/kg DM) and vitamin and mineral premix (47 g/kg DM; Multitec of Malta^®^, Guanajuato, Mexico). Chemical composition of ingredients used in the experimental diets is shown in Table 1.

Sunflower seed silage was prepared as follows: 50 kg of sunflower seeds were crushed into 2 mm particles. The next step was the addition of water 1:1 ratio and 0.001% fresh *Pulque* as an inoculant to accelerate the fermentation process [9]. *Pulque* is a Mexican fermented beverage readily available around the region where the study was carried out, which is rich in lactic acid bacteria (1.5 × 10^8^ CFU/mL), aerobic mesophilic bacteria (1.2 × 10^7^ CFU/mL), and yeast (1.9 × 10^7^ CFU/mL) [10]. After *Pulque* inoculation, the crushed seeds with water and *Pulque* were homogenized (pre-silage). Then, placed in layers, compacted, sealed, and ensiled in 25-kg plastic bags (n = 4). The plastic bags were placed hard-plastic containers (975 mm height, 594 mm Ø, and 208 L of capacity) with a hard cover, and sealed to avoid rodents and birds presence. Silages were fermented for 24 days, until their use for feeding animals. This silage-making method is similar to conventional methods used for corn silage-making; however, in this case we used a local fermented beverage as a natural inoculant.

**Table 1:** Chemical composition (g/kg DM) of ingredients included in the dietary treatments formulation.

Ingredient	Corn Silage	Alfalfa Hay	Sorghum Gain	Triticale Grain	Soybean Meal	SF	SFS
Dry matter	270	900	932	900	923	927	553
Organic matter	939	900	919	934	905	961	955
Crude protein	84	180	80	123	443	205	257
Ether extract	16.8	30	26.7	25	11.9	281	378
Neutral detergent fiber	545	550	46	231	70	408	331
Acid detergent fiber	322	330	23	64	37	333	238
ME, MJ/kg DM ^1^	11	10	13	13.2	13.6	17.5	17.5

^1^ Calculated from NRC [11]. SF = crushed sunflower seed; SFS = sunflower seed silage; ME = metabolizable energy.

Dietary treatments were elaborated to satisfy energy and protein requirements of dairy ewes in mid-lactation [11]. Dietary treatments were balanced to theoretically contain 115 g/kg of crude protein and 10.04 MJ of metabolizable energy (ME)/kg DM [11].

The concentrate was supplied twice daily at 08:00 and 16:00 hours. Depending on the treatment, amounts of concentrate per animal per day were: 752 g for control, 798 g for SF, and 869 g for SFS. The concentrate was made in batches of 100 kg by manually mixing the ingredients for each diet. Concentrate and forages were offered separately as each individual stall had a feed bunk divided in two spaces. Chemical composition of the dietary treatments is shown in Table 2.

### 2.2. Sampling and Measurements

In each experimental period, samples of corn silage, alfalfa hay, concentrate, and treatments were collected every day and stored at −20 °C. Dietary treatments, orts, and fecal samples were dried in a forced-air oven at 60 °C for 48 h. Once dried, they were ground with a Wiley mill (2.0 mm screen; Arthur H. Thomas, Philadelphia, PA, USA), and analyzed in duplicates for DM (930.15), organic matter (OM; 942.05), ether extract (method 920.39) and nitrogen (N; 990.02) using the Association of Official Analytical Chemists (AOAC) [12] standard methods. Neutral detergent fiber (NDF) and acid detergent fiber (ADF) were determined following Van Soest et al. [13] methods. Nutrient digestibility (g/kg) was determined as [(nutrient intake, g/d – nutrient excreted, g/d)/(nutrient intake, g/d)] × 1000.

Feces were collected every 24 h and sampled at 08:00 h on the last 6 days of each period. Feces were collected from each individual stall that was equipped with a metallic container with a mesh frame. Feces were fully collected and used for calculations. A subsample of 10% was taken and stored at −20 °C until analysis.

Urine was collected every 24 h and sampled every day at 08:00 h during the last 6 days of each period. Urine samples were obtained using a metal container located under each individual stall that had a mesh filter screen to allow separation of urine from feces. Daily total volumes of urine were measured and then 10% of total samples was kept and frozen at −20 °C for further analysis. Feces and urine samples were used to quantify nitrogen excretion. Total nitrogen in feces and urine was analyzed using micro-Kjeldahl analysis.

Individual body weight (BW, kg) and body weight change (BWC, g/d) were registered at the beginning and at the end of each experimental period. Dry matter intake (DMI, g/d) and individual milk yields (kg/d) were recorded every day but only data from the last 6 days of each period were used for statistical analysis.

Individual milk samples (100 mL) were collected on the last 6 days of each experimental period at 16:00 h using a volumetric milk meter and preserved with potassium dichromate (Merck, Fontenay-sous-Bois, France). Milk samples were analyzed in duplicates by infrared using a MilkoScan 133B (Foss Electric, Hillerød, Denmark) in order to determine total solids (TS) and non-fat solids (NFS). Milk protein was determined according to McKenzie and Murphy [14] and fat was determined by Levowitz method [15]. Milk urea nitrogen (MUN) was analyzed by the micro-Kjeldahl method, and the protein content was determined using a factor of 6.38 based on method 991.20 [12].

Fat-corrected milk (FCM 6.5%, kg/d) and fat-protein corrected milk (FPCM 6.5, 5.8%, kg/d were calculated according to Pulina et al. [16]. Feed efficiency (FE) was calculated as: FE = milk yield (kg/d)/dry matter intake (kg/d). Adjusted FE was calculated as [6.5% FCM (kg/d)/DMI (kg/d)].

### 2.3. In Vitro Gas Production

Each dietary treatment and pure ruminal fluid were used to determine in vitro gas production [17]. A total of 0.800 g DM of each dietary treatment was put into glass flasks bottles by triplicate in two tandems and repeated for four incubation runs (total of 24 bottles of each diet and treatment), with 90 mL of buffer solution and 10 mL of sheep rumen fluid. The buffer solution composition has been reported previously [18]. Three sheep (42 ± 2 kg of live weight) from the same herd fed on the control diet were used as donors of ruminal fluid, which was extracted, filtered in triple cheesecloth gauze, and homogenized with CO_2_. The bottles were incubated in a water bath at 39 °C. The volume of gas (ml of gas/g DM) was recorded at 3, 6, 9, 12, 24, 36, 48, 60, 72, 84, and 96 h of incubation. The gas accumulated from each sample was adjusted to the model proposed by France et al. [19]. After 96 h of incubation period, pH and the dry matter disappearance (DMD) were determined. A completely randomized design was performed, and Tukey’s test was used when differences between treatments were observed.

### 2.4. Statistical Analysis

Data from the last 6 days of each experimental periods were considered for statistical analysis. Data were subjected to ANOVA using the general linear model procedure of the SAS software package (2002), with a model that included the effects of ewe, period, and treatment in a Latin square design, where experimental period and treatment were considered fixed effects, and individual ewe the random effect. Least squares means with their standard errors are reported and significant treatment effects were declared at *p* < 0.05.

## 3. Results and Discussion

### 3.1. Nutrient Intake and Digestibility

The live weight remained similar between treatments (52.3 ± 4.90 kg) (Table 3). This was expected as diets had similar energy content and the dry matter intake did not change across treatments. Khotija et al. [20] suggested that the addition of sunflower seed is an efficient way to supply energy and that it helped to maintain the body conditions of postpartum lactating ewes, as observed in this study. Conversely, Ivan et al. [21] found a significant decrease from 240 g to 191 g in daily weight gains in growing lambs with the addition of crushed sunflower seed at 14% per kg DM in a high forage diet (50% DM), and that was attributed to a decrease in DMI in response of the low digestibility of the forage. In the same study, but with a low dietary forage and protein (crushed sunflower seed), the average daily gain was higher due to the lower fiber content, even with a decrease DMI against control.

Both SF and SFS had no effect on DM (1124 ± 85.7 g/d) and OM (1781 ± 182 g/d) intakes (Table 3), which partly agrees with what was previously reported both in lactating sheep [20,22,23] and goats [24,25], when animals were fed with either sunflower seeds or sunflower oil. Gomez-Cortés et al. [23] obtained a rise in DM intake when sheep were supplemented with 2% DM sunflower oil in a diet, with a diet based on 50% forage and 50% concentrate; however, contrary to the present results, Ivan et al. [21], reported a decrease in DMI with low- and high-forage diets with two protein levels of crushed sunflower seed supplementation.

Compared to control, SF and SFS improved intake and digestibility of NDF and ADF (Table 3), while the SFS treatment obtained the highest digestibility of NDF and ADF. This was probably related to the toxic effect of polyunsaturated fatty acids (which are high in sunflower seeds) on the predatory ciliated protozoa of beneficial ruminal fauna (defaunation) that led to increase the populations of cellulolytic bacteria, improving digestibility of NDF and ADF. This effect was described previously in growing lambs fed on sunflower seeds [21].

### 3.2. Nitrogen Balance

Nitrogen balance was higher in ewes fed SF and SFS diets, while SFS increase milk N retention (Table 4). The amounts of N in feces (6.77 ± 0.93 g/d) were of higher magnitude than that observed in urine (3.65 ± 0.59 g/d), which suggests that there was a larger use of ruminal ammonia, causing a transfer of N from urine to feces [26].

In this study, the absence of changes in nitrogen utilization was expected as dietary treatments were balanced to be isonitrogenous. In ruminant diets, energy supply is important for rumen microbial growth and has a deep impact on protein metabolism [27].

The positive N balance found with crushed sunflower seeds and sunflower silage points at the ability of both feedstuffs to deliver the required nitrogen for lactating ewes, while fulfilling protein needs for rumen microorganisms [11]. The oil contained in both SF and SFS diets did not affect the growth of cellulolytic bacteria, as suggested by the data about digestibility of NDF (Table 3). This probably favored a more efficient use of the ammonia originated by the rumen degradable protein of the SF and SFS diets. Our results also coincide with a positive N balance reported in lactating does fed whole sunflower seeds or linseed [24].

### 3.3. Milk Yield and Milk Components

Milk yield (0.68 ± 0.14 kg/d), milk fat yield (38.4 ± 9.12 g/d) and milk protein yield (30.1 ± 6.32 g/d) were not affected by treatments (Table 5). In sheep, concentrate-rich diets are expected to increase milk yields; however, that may lead to detrimental effects on lipid metabolism [28]. In goats fed on a corn silage-based diet and supplemented with sunflower oil, Bernard et al. [29] reported a rise in milk yield compared to their control treatment (no additional oil). However, in goats fed on a grass silage-based diet supplemented with whole seeds of sunflower or linseed, Vargas-Bello-Pérez et al. [24] did not find changes in milk yield and milk composition. Based on those studies, in the present study, the forage to concentrate ratio was around 57:47, and the lack of differences in milk production are partly explained by the amount of dietary forage that was supplied to the animals as ad libitum corn silage. Compared to concentrate-based diets, forage-based diets provide greater amounts of fiber (NDF and ADF) and lower non-structural carbohydrate content, such as sugars and starch [30] and this was reflected in our study.

Compared with control and SF, SFS increased contents (g/100g) of protein, lactose, and non-fat solids (Table 5). These results suggest that SFS was able to supply enough N for rumen microbial protein synthesis and this was later revealed in the secreted milk. This finding on milk protein contents reinforce the observed positive N balance that was earlier discussed in this manuscript. Moreover, the increase in milk protein by SFS may be due to the fact that this treatment may have been able to provoke rumen defaunation of ciliated protozoa and that led to increases in rumen microbial synthesis of protein [21].

Milk fat content (5.42 ± 0.43 g/100g) and yield were similar between treatments (Table 5). Compared with cows, sheep and goats are more resilient to dietary supplements rich in polyunsaturated fatty acids, such as oilseeds by-products. In this study, we fed animals with two types of sunflower seed presentation (crushed and ensiled) and in both cases they did not induce milk fat depression. One explanation to the different responses to dietary lipids between ruminant species is that there are differences in their rumen bacterial structure, especially in goats [31]. However, when diet provides enough amount of effective fiber, no reductions in milk fat have been observed in both small and large ruminants [32,33,34,35]. According to Chilliard et al. [36], if the concentrate does not exceed 60% of the diet, the milk fat content is not much affected, whereas a significant decrease is often observed beyond 60%. In the present experiment, SF and SFS diets were associated with higher intake of NDF that probably avoid detrimental effect of the highest content of dietary unsaturated fatty acids on rumen cellulolytic bacteria. Future studies should analyze the effects of SF and SFS on milk fatty acid profile as today consumers are more aware on the benefits and disadvantages from consuming milk fat [37], and in the case of the present study, it will be expected to obtain reductions in fatty acids derived from de novo synthesis and odd- and branched- chain fatty acids [38].

### 3.4. In Vitro Gas Production

The fractional rate of degradation (c) and lag time were not affected by treatments (Table 6). Gas production at 12 and 24 h was higher for SFS. Compared with control, SF and SFS decreased DM disappearance at 96 h. Although no pure sunflower oil was used in this study, it is possible that the oil contained in the sunflower seeds either crushed or ensiled, was sufficient to disrupt the rumen microbial ecosystem as fat coats feed particles, affecting microbial fermentation [39].

Compared with control and SF, microbial crude protein production was reduced by SFS (Table 6). Normally, fat, oils, and grease are related to diminished microbial activity [40] and this was reflected in the reductions of microbial crude protein provoked by both types of sunflower presentations. These results are in contrast with in vivo data obtained from the present study, probably due to the use of “in batch” fermentation. According to the in vivo results, NDF and ADF digestibility was greater in SF and SFS diets, and, likely, the microbial protein production was also enhanced, as suggested by the higher N retention. Ewes fed SF and SFS diets increased concentrate and NDF intake per kg of live weight, due to the higher NDF content of SF and SFS that, in turn, likely stimulated the passage rate of diet ingredients. This likely reduced the inhibitory effect of vegetable oil on cellulolytic bacteria. When fermentation was investigated by “in batch” system, the effect of passage rate was not evaluable. To support our findings, future studies should consider analyzing in detail in vitro rumen simulation technique (i.e., RUSITEC system) or in vivo feeding trials focused on rumen microbiome.

## 4. Conclusions

Overall, our results highlight the importance of the basal diet composition on responses related to milk yield, milk composition, nutrient degradation, nutrient intake, and N balance in dairy sheep. Results demonstrated that crushed sunflower seeds and ensiled seeds do not change significantly productive parameters of dairy sheep. In corn-silage based diets, both crushed and ensiled sunflower seeds, could be used in dairy sheep diets as alternatives for protein feedstuffs.

## Figures and Tables

**Table 2:** Inclusion of ingredients (g/kg DM) and chemical composition (g/kg DM) of control, sunflower seeds (SF) and sunflower seeds silage (SFS) treatments.

Ingredients	Treatments		
	Control	SF	SFS
Corn silage	*Ad libitum*	*Ad libitum*	*Ad libitum*
Alfalfa hay	333	333	333
Sorghum grain	253	267	267
Triticale grain	200	100	100
Soybean meal	167	167	167
Crushed sunflower seeds	0	87	0
Sunflower seed silage	0	0	87
Vitamin and mineral premix ^1^	47	47	47
Chemical composition			
Dry matter	905	928	903
Organic matter	870	873	872
Crude protein	179	185	190
Ether extract	24	46	54
Neutral detergent fiber	253	266	259
Acid detergent fiber	135	158	149
Metabolizable energy, MJ (kg DM ^2^)	11.5	11.9	11.9

^1^ Containing in 1.0 kg DM the following: 25 mg of antioxidant, 4.5 g of calcium carbonate, 6 g of salt, 30 g of ionophore, 50 g of zinc oxide, 6 g of sodium bicarbonate, 6 g of copper sulfate, 20 g of ferrous sulfate, 125 g of sodium sulfate, 18,000 IU of vitamin E, 3,000,000 IU of vitamin A, 3,750,000 IU of vitamin D, 140 g of potassium chloride, 0.500 g of EDD. I ethylene-dynamine, 0.090 g of cobalt carbonate, 500 mg of magnesium oxide, 36 g of manganese oxide and 0.090 g of selenium. ^2^ Calculated from NRC [11].

**Table 3:** Intake (g/d, g/kg LW^0.75^) and digestibility coefficients (kg/kg) in dairy sheep fed control, sunflower seeds (SF), and sunflower seeds silage (SFS) treatments.

	Treatment			SEM	*p*-Value
	Control	SF	SFS		
Average body weight, BW, kg	52.5	51.9	52.5	4.90	0.853
Average metabolic BW, g/kg LW^0.75^	19.4	19.2	19.4	1.36	0.995
Intake, g/d					
Dry matter	1127	1100	1144	85.7	0.981
Forage:concentrate ratio	1.37 (57:43)	1.47 (59:41)	1.21 (55:45)	0.13	0.400
Organic matter	1683	1860	1799	182	0.975
Crude protein	251	297	318	25.5	0.199
Fat	66 ^b^	90 ^ab^	124 ^a^	12.6	0.017
Neutral detergent fiber	530 ^b^	833 ^a^	788 ^ab^	78.6	0.033
Acid detergent fiber	259 ^b^	475 ^a^	449 ^a^	43.8	0.006
Intake, g/kg LW^0.75^					
Dry matter	92.0	102	98.1	5.32	0.419
Forage intake	53.3	60.9	53.7	5.21	0.524
Concentrate intake	38.7 ^b^	41.3 ^ab^	44.3 ^a^	0.84	0.001
Sunflower intake	0.00 ^c^	5.00 ^b^	7.82 ^a^	0.69	0.001
Organic matter	85.8	96.0	92.2	4.95	0.362
Crude protein	12.9 ^b^	15.3 ^a^	16.2 ^a^	0.39	0.001
Fat	3.41 ^b^	4.65 ^b^	6.24 ^a^	0.34	0.001
Neutral detergent fiber	26.8 ^b^	43.0 ^a^	40.4 ^a^	2.32	0.001
Acid detergent fiber	13.1 ^b^	24.5 ^a^	23.0 ^a^	1.28	0.001
Digestibility coefficient, kg/kg					
Dry matter	720	700	720	15.5	0.497
Organic matter	740	730	750	12.2	0.571
Crude protein	840	850	860	11.2	0.547
Neutral detergent fiber	560 ^b^	650 ^a^	700 ^a^	14.6	0.001
Acid detergent fiber	370 ^b^	600 ^a^	620 ^a^	17.0	0.001

^a,b,c^ Different letters indicate significant differences (*p* < 0.05). SEM = standard error the mean. Forage:concentrate ratio = forage (alfalfa hay and corn silage), concentrate (sorghum grain triticale grain, soybean meal, sunflower seeds (crushed or ensiled) and vitamin and mineral premix).

**Table 4:** Nitrogen balance (g N/d, g N/kgLW^0.75^) in dairy sheep fed control, sunflower seeds (SF), and sunflower seeds silage (SFS) treatments.

N Balance	Treatment			SEM	*p*-Value
	Control	SF	SFS		
N intake, g/d	40.2	47.6	51.0	4.08	0.199
N intake, g/kg LW^0.75^	2.06 ^b^	2.46 ^a^	2.60 ^a^	0.06	0.001
Fecal N excretion g/d	6.37	7.20	6.76	0.93	0.819
Fecal N g/kg LW^0.75^	0.32	0.37	0.34	0.03	0.597
Fecal N % of N	15.3	15.0	13.3	1.23	0.474
Urine N excretion g/d	2.69	3.54	4.72	0.59	0.087
Urine N g/kg LW^0.75^	0.14	0.18	0.25	0.04	0.201
Urine N % of N	7.14	7.64	10.0	1.89	0.537
Milk N excretion g/d	9.93	11.2	8.27	2.19	0.630
Milk g/kg LW^0.75^	0.48	0.57	0.40	0.08	0.382
Milk % of N	22.8	23.2	15.1	3.53	0.220
Milk N retention g/d	21.1 ^b^	24.5 ^b^	29.7 ^a^	2.06	0.031
N balance g/kg LW^0.75^	1.59 ^b^	1.90 ^a^	2.00 ^a^	0.07	0.004
% Retained N	54.0	51.8	58.6	3.74	0.448

^a,b^ Different letters indicate significant differences (*p* < 0.05). SEM = standard error the mean.

**Table 5:** Milk yield and milk composition from dairy sheep fed control, sunflower seeds (SF), and sunflower seeds silage (SFS) treatments.

Item	Treatment				
	Control	SF	SFS	SEM	Treatment
Milk Yield, kg/d	0.64	0.78	0.62	0.14	0.695
Fat-corrected milk 6.5%	0.63	0.72	0.52	0.14	0.603
FPCM 6.5, 5.8%	0.60	0.69	0.52	0.13	0.647
Feed Efficiency	0.32	0.35	0.26	0.05	0.359
Feed Efficiency FCM	0.30	0.34	0.25	0.04	0.404
Milk-N/ N-Intake%	0.22	0.27	0.22	0.03	0.503
MUN, mg/dl	74.8	95.4	105	11.4	0.199
Milk composition, g/100g					
Fat	5.78	5.69	4.79	0.43	0.810
Protein	4.23 ^b^	4.38 ^ab^	4.58 ^a^	0.10	0.043
Lactose	4.00 ^b^	4.15 ^ab^	4.34 ^a^	0.10	0.042
Non-fat solids	8.93 ^b^	9.29 ^ab^	9.72 ^a^	0.22	0.027
Total solids	22.9	23.5	23.4	0.56	0.752
Milk composition, g/d					
Fat	40.7	44.1	30.5	9.12	0.541
Protein	26.4	34.8	29.0	6.32	0.468
Lactose	25.0	32.9	27.5	5.98	0.466
Non-fat solids	55.9	73.9	61.9	13.4	0.450
Total solids	148	185	149	34.2	0.680

^a,b^ Mean values for each experiment within a row with unlike superscript letters were significantly different (*p* < 0.05). SEM = standard error the mean. Fat-corrected milk (FCM 6.5%, kg/d) = [milk (kg/d) × (0.37 + fat %) × 0.097], Fat-Protein corrected milk (FPCM 6.5, 5.8%, kg/d) = [milk (kg/d) × (0.25 + 0.085 fat% + 0.035 protein%)], Feed Efficiency = Milk (kg/d)/DMI (kg/d), Adjusted Feed Efficiency FCM6.5% = FCM6.5% (Kg/d)/DMI (kg/d). Milk-N/ N-Intake% (Milk N, kg/d/ N intake, kg/d × 100,) MUN, mg/dL = milk urea nitrogen.

**Table 6:** In vitro rumen gas kinetics (mL gas/ g DM) and fermentation profile of different diets in dairy sheep fed control, sunflower seeds (SF) and sunflower seeds silage (SFS) treatments.

Item	Control	SFS	SFS	SEM	*p*-Value
A	186	183	174	5.08	0.298
B	0.044	0.038	0.050	0.008	0.665
C	0.002	0.003	0.012	0.017	0.903
Lag time	0.258	0.352	0.218	0.079	0.931
Gas production, mL gas/g DM					
12 h	85 ^ab^	77 ^b^	90 ^a^	2.56	0.001
24 h	124 ^a^	112 ^b^	122 ^a^	2.73	0.001
48 h	158 ^a^	152 ^b^	150 ^b^	2.61	0.001
96 h	188 ^a^	181 ^b^	176 ^b^	3.03	0.001
pH	6.78	6.79	6.77	0.02	0.685
DMD 96 h	84.9 ^a^	83.2 ^b^	83.0 ^b^	0.30	0.007
PF 96 h	221	218	212	4.12	0.326
GY 24 h	29.2	26.9	29.4	0.62	0.057
SCFA	0.55	0.49	0.54	0.01	0.056
MCP	794 ^a^	782 ^ab^	776 ^b^	2.79	0.001

^a,b^ Mean values for each experiment within a row with unlike superscript letters were significantly different (*p* < 0.05). SEM = standard error the mean. A = total gas production (ml gas/g DM incubated); B = fermentation rate (h^−1^); C = fermentation rate (h^−1/2^); Lag time = the initial delay before gas production begins (h); DMD96 = DM degraded substrate (mg/g DM); GY24 = gas yield at 24 h (mL gas/g DMD); SCFA = short chain fatty acids (mmol/g DM); MCP = microbial CP production (mg/g DM).
